# Price elasticity of cigarette smoking in Bangladesh: evidence from the Global Adult Tobacco Surveys (GATS)

**DOI:** 10.1136/tc-2022-057668

**Published:** 2023-08-08

**Authors:** Rumana Huque, S M Abdullah, Md Nazmul Hossain, Nigar Nargis

**Affiliations:** 1 Department of Economics, University of Dhaka, Dhaka, Bangladesh; 2 Research, ARK Foundation, Dhaka, Bangladesh; 3 Economic and Health Policy Research, American Cancer Society, Washington, District of Columbia, USA

**Keywords:** Price, Taxation, Economics, Socioeconomic status, Low/Middle income country

## Abstract

**Introduction:**

The overall prevalence of cigarette smoking has not changed significantly for over a decade in Bangladesh. Raising the price of cigarettes through taxation is an important policy instrument for reducing consumption and achieving public health goals. The price elasticity of cigarette demand is an important parameter for evaluating the effectiveness of raising prices through tax increases in reducing cigarette consumption. The objective of the study was to estimate the price elasticity of cigarette demand in Bangladesh using Global Adult Tobacco Survey 2009 and 2017 data.

**Methods:**

Smoking prevalence and smoking intensity were estimated using a two-part model. Endogeneity of prices was minimised using the average consumption-weighted cigarette price in a cluster, for both smokers and non-smokers residing in a specific cluster.

**Results:**

Cigarette demand was found to be price inelastic and ranged between −0.51 and −0.73. It is also price inelastic across wealth groups and areas of residence in Bangladesh. Although the total price elasticity did not differ considerably between rural and urban locations, it is evident that individuals in the lower-wealth group are more than twice as responsive to price increases as their high-wealth counterparts.

**Conclusion:**

A significant increase in cigarette prices through a tax increase would decrease smoking prevalence and increase tax revenue in Bangladesh. The greater price sensitivity among smokers in lower-wealth groups indicates that a tax-induced cigarette price increase would provide more health benefits to them, thereby contributing to improved health equity.

**WHAT IS ALREADY KNOWN ON THIS TOPIC**

- Cigarette demand responds less than proportionately to price increases and hence is price inelastic.

**WHAT THIS STUDY ADDS**

- This is the first study that has estimated the overall impact of price changes on the prevalence and intensity of cigarette smoking in Bangladesh, using Global Adult Tobacco Survey data.
- The estimated price elasticity ranged between −0.51 and −0.73. Individuals in the low-wealth group are more than twice as responsive to price change as their high-wealth counterparts.
- Biri price is not a key determinant in the intensity of cigarette smoking. The weak substitutability of cigarettes and biris suggests that cigarette smokers are unlikely to switch to biris in response to price increases.

**HOW THIS STUDY MIGHT AFFECT RESEARCH, PRACTICE OR POLICY**

- Substantial increase in cigarette prices at the low tier would effectively reduce cigarette consumption among people from low-wealth groups in Bangladesh.

## Introduction

Bangladesh has one of the largest tobacco-using populations (37.8 million adults; prevalence 35.3%) in the world.^1^ The cigarette smoking prevalence among adults in Bangladesh has remained steady at around 14% over the last decade. Among males, the prevalence was 28.3% in 2009 and 28.7% in 2017, while among females, it was stagnant at around 0.2% in 2009 and 2017.^1 2^ Cigarette prevalence also varied considerably across income groups, educational attainment and location. It is highest among men with no formal education (35.6%), belonging to the lowest wealth quintile (30.7%) and living in urban areas (31.5%).^1^

Taxation is an important price instrument for regulating the consumption of tobacco products.^3 4^ While increasing the price of tobacco products through taxation helps to reduce tobacco use, the magnitude of the response is not uniform. The effectiveness of a tax increase depends on both the increase in price resulting from the tax change and the price elasticity of demand (ie, the responsiveness of quantity demanded to a change in price), among other factors. The total price elasticity of cigarettes can be further divided into prevalence and intensity elasticity. Prevalence elasticity measures the proportionate change in the decision to smoke cigarettes due to the percentage change in price, while intensity elasticity measures the percentage change in the number of sticks a smoker smokes when the price changes.

Cigarette price elasticity estimates varied across countries and over time. In a recent cross-country study, total cigarette price elasticity for 13 low and middle-income countries (LMICs) was estimated to be −0.53, with a prevalence and intensity elasticity of −0.36 and −0.17, respectively.^5^ For LMICs in the Asia-Pacific region, it was −0.35.^6^ Country-specific estimates in LMICs (eg, Estonia, Turkey, China, Myanmar, Bulgaria, Egypt, India, Indonesia, Malaysia, Jordan, Mexico, South Africa, Sri Lanka, Thailand, Turkey, Ukraine and Vietnam) fell between −0.50 and −1.05 and clustered around −0.50.^3 7–29^ Several estimates for cigarette price elasticity are available in Bangladesh. Ali *et al*, Guindon *et al* and Barkat *et al* used time-series data to estimate the price elasticity of cigarettes.^30–32^ The former two found price to be a statistically insignificant determinant for cigarette consumption, while the latter estimated price elasticity as −0.41 and −0.57 in the short run and long run, respectively. Several others used cross-sectional data for this purpose. Examples include Nargis *et al*, Del Carmen *et al* and Ahmed *et al*.^33–36^ Nargis *et al* 34 concluded that cigarettes are price inelastic, while the other two found them to be price elastic.^33–36^ Unlike the others, Nargis *et al* 34 separated the price elasticity of cigarettes into prevalence and intensity elasticity.^33–36^

The tobacco-control policy environment in Bangladesh changed considerably over the decade from 2009 to 2017, including the amendment to the Tobacco Control Act 2013 and subsequent strengthening of non-tax tobacco-control measures, which might have interacted with price changes to affect cigarette demand. Previous studies estimating the cigarette price elasticity in Bangladesh had limitations due to the omission of this effect. The present study improves upon previous estimates by pooling observations from 2009 and 2017 and accounting for the time effect, capturing the shift in tobacco-control policy environment in isolation from the tax and price changes for cigarettes, and thus takes a more comprehensive approach to estimate the cigarette price elasticity. In addition to the overall measure of elasticity, this study estimated the elasticities of cigarette smoking by region to compensate for its absence in the existing literature for Bangladesh.

## Methods

Although cigarette is addictive, its consumption follows the basic demand law. Estimation of the cigarette demand function requires an assumed specification of a consumer utility function that includes utility from cigarette consumption along with other goods. Consumers maximise utility subject to a budget constraint determined by their income, the price of cigarettes and of other goods. Given the price and income, there can be a corner solution of zero consumption for those who decide not to smoke and an interior solution of positive consumption for those who decide to smoke. Empirically, these can be modelled using two approaches—a ‘sample selection model’ and a ‘two-part model’.^37–39^ The choice of one over the other depends on whether the outcome of interest is potential or actual.^40^ While the sample selection approach models the potential outcome in stated preference, the two-part approach is suitable for modelling actual outcomes that indicate revealed preference.^41^ Considering the mix of non-smokers (corner solution with zeros) and smokers (interior solutions with positive integers) in the study sample, a two-part approach was applied. Two equations were estimated separately: smoking propensity in the first part and smoking intensity, conditional on smoking participation, in the second. Since the two equations were estimated sequentially and independently of each other, the possibility of simultaneous determination of smoking propensity and intensity was ruled out. We have, however, examined the extent of independence using the correlation among residuals in both equations and confirmed that the magnitude was negligible.

### First part: modelling smoking prevalence (smoking decision or propensity)

The decision to smoke and, hence, cigarette consumption, respond negatively to price increases.^5 6 42–45^ Accordingly, price of cigarettes (P_c_) is the independent variable and cigarette smoking prevalence is the outcome in the first part. Additionally, the price of other substitutable tobacco products, such as biri (P_B_), and individual socioeconomic and demographic attributes (income (I), age (A), gender (female) (G_F_), residential area (rural) (R_R_), educational attainment (EA), employment category (EC) and family size (FS)) are important in determining smoking prevalence. Furthermore, smoking restrictions in home (SR_H_) and workplace (SR_W_) discourage smoking in order to reduce exposure to secondhand smoke. Similarly, the provision of health warnings (W), advertising (Ad), promotional activities (P_RM_) and individuals’ smoking perception (SP) can affect smoking uptake. Therefore, a probit regression that models the inverse standard normal distribution of the probability of smoking as a linear combination of the predictors was used:

(1) $$P_{r}\left(CigaretteSmoking>0\right)=\begin{array}{l} \Theta \left(\beta_{0}+\beta_{1}P_{c}+\beta_{2}P_{B}+\beta_{3}I+\beta_{4}A+\beta_{5}FS+\beta_{6}G_{F}+\beta_{7}R_{R}+\;\sum \beta_{8i}EA_{i}+\sum \beta_{9i}EC_{i}+\beta_{10}SR_{H}+\beta_{11}SR_{W}+\sum \beta_{12i}W_{i}+\;\sum \beta_{13i}Ad_{i}+\sum \beta_{14i}P_{RMi}+\sum \beta_{15i}SP_{i}+\varepsilon_{i}\right) \end{array}$$

where $\varepsilon_{i}$ denotes the disturbance term. Except P_C_, P_B_, A and FS, all other covariates were categorical and took a dummy approach. Subject to exposure to an event (eg, smoking restrictions, warnings, advertising, promotional activities), individuals were coded as 1, and 0 otherwise. The probit model was chosen as it had higher log-likelihood than other probability models and has been widely used for tobacco demand modelling.^34 46–49^ The price elasticity of cigarette smoking prevalence was estimated using the following formula:

(2) $$PrevalenceElasticity=\theta \left(.\right)\beta_{1}\times \frac{AverageConsumptionWeightedCigarettePrice}{PopulationProbabilityofCigaretteSmoking}$$

In equation 2, θ(.) is the normal density evaluated at the average values of covariates and $\beta_{1}$ is the marginal effect of price changes in equation (1).

### Second part: modelling smoking intensity (number of cigarettes smoked per day)

In part two, conditional on an individual being smoker, the number of cigarettes smoked was modelled using the following stochastic log-linear regression equation:

(3) $$lny=\alpha_{0}+\alpha_{1}P_{c}+\alpha_{2}P_{B}+\alpha_{3}I+x^{\prime }\alpha_{4}+\varepsilon ,where,y>0$$

Here, *lny* indicates the natural logarithm of the average number of sticks smoked by a person and *x* is the vector of socioeconomic and demographic covariates used in first part. The marginal effect of price on cigarette smoking intensity in equation 3 depends on the value of the average number of sticks smoked (*y*) as (*1/y)(dy/dP_c_)=α_1_* or *dy/dP_c_= α_1_×y*. The price elasticity of smoking intensity is measured using the following formula:

(4) $$IntensityElasticity=\frac{dy}{dPc}\times \frac{Pc}{y}=\alpha_{1}\times AverageConsumptionweightedCigarettePrice$$

The total elasticity is the summation of elasticities from the first and second parts with respect to price.

(5) TotalPriceElasticityofCigarette=PrevalenceElasticityofCigarette+IntensityElasticityofCigarette

### Data, measures and specifications

The Global Adult Tobacco Survey (GATS) 2009 and 2017 data from Bangladesh were used. GATS is a nationally representative survey that provides self-reported information on price and smoking behaviour, along with individual and household characteristics. A total of 9629 and 12 783 randomly selected individuals were surveyed in GATS 2009 and GATS 2017, respectively.^1 2^ The estimation sample pooled the two rounds of GATS and the time effect was controlled for during estimation. The analytical sample was reduced to 11 471 individuals (5282 from GATS 2009 and 6189 from GATS 2017), particularly because of insufficient observations of the biri price. The outcome, that is, cigarette smoking prevalence, contained two groups: cigarette smokers (smoked cigarettes daily or less than daily in the last 12 months) and non-smokers (do not smoke at all currently). The unitary cigarette prices were calculated by dividing the cigarette expenditure in the last purchase by the corresponding quantity of purchase. Prices were calculated per stick instead of per pack due to the high volume of single-stick cigarette purchases (80.82% of purchases were single sticks, estimated using GATS 2017 in Bangladesh).^50^ Real prices were estimated using the Consumer Price Index of 2017.^51^

Given the unavailability of income data in GATS, asset holding status (eg, whether a household has electricity, phone, television, refrigerator, car/motorbike/bicycle, washing/sewing machine, table/chair/bed/almirah, watch/clock) was used to construct a wealth index as a proxy for permanent income.^52^ Asset weight was estimated using Principal Component Analysis.^53 54^ The index was generated considering the asset availability and particular weight (online supplemental file contains the details).

10.1136/tc-2022-057668.supp1 Supplementary data

Three different models were estimated. In model 1, cigarette smoking prevalence and smoking intensity were modelled with respect to P_c_, P_B_, and I (proxied by wealth index); model 2 augmented this basic model by including demographic (A, FS, G_F_ and R_R_) and socioeconomic variables (EA and EC); and model 3 augmented models 1 and 2 by including SR_H_, SR_w_, W, Ad, P_RM_ and SP. The model specifications were tested using link test.^55^ While we estimated the models separately for 2009 and 2017 (online supplemental file contains the estimation), the pooled estimates were reported for two reasons. First, the prevalence of cigarette smoking did not change significantly over this period in Bangladesh, while cigarette prices changed considerably. We preferred to exploit both cross-sectional and historical variations in cigarette prices, adjusted for inflation over time, to identify the effect of price changes on individual cigarette smoking behaviour. Second, significant policy changes took place during this period, notably the introduction of the Health Development Surcharge, and these needed to be adjusted to identify the effect of price variation on individual cigarette smoking behaviour. In the pooled analysis, we used the time effect that controlled for concomitant changes in tobacco-control policies as well as the overall macroeconomic environment. Results from separate estimations of the model for the individual years are limited by the lack of adjustment for time effect.

### Endogeneity of cigarette and biri price

Smokers’ self-reported prices might have endogeneity. To minimise this, a consumption-weighted price for each cluster was assigned to all individuals residing in the cluster. The weight for a smoker was defined as the relative share of his or her cigarette consumption in the total consumption of cigarettes within the cluster. The weighted price was calculated by multiplying the self-reported per-stick price with the corresponding consumption weight. The cluster average of consumption-weighted prices was assigned to both smokers and non-smokers residing in a specific cluster. For determining the price of biri, a similar procedure was followed.

## Results

### Overall data

Table 1 presents the price elasticity estimates of the prevalence and intensity of cigarette smoking in Bangladesh. Corresponding marginal effects for the models are presented in online supplemental table 1. Estimates of prevalence elasticities in all three models reveal that cigarette price significantly affects the decision to smoke cigarettes. In model 3, the estimate was −0.69, implying that a 10% increase in the price of a cigarette stick would lead to a decrease in the prevalence of cigarette smoking by 6.9% on average. The price elasticity was estimated to be 4.5% in model 1 and 6.7% in model 2. The results confirmed that cigarette smoking intensity is also affected negatively by cigarette price. In model 1, the intensity elasticity was estimated as −0.06. It indicates that a 10% increase in price would lead to an average 0.6% reduction in smoking intensity. However, the effect of price on smoking intensity was statistically insignificant in the other two models.

**Table 1:** Prevalence and intensity elasticity of cigarette smoking using GATS data

Variables	Prevalence elasticities			Intensity elasticities		
	Model 1	Model 2	Model 3	Model 1	Model 2	Model 3
Average consumption-weighted cigarette price (BDT per stick)	−0.45*** (0.09)	−0.67*** (0.14)	−0.69*** (0.14)	−0.06** (0.03)	−0.04 (0.03)	−0.04 (0.03)
Average consumption-weighted biri price (BDT per stick)	−0.01 (0.01)	−0.03* (0.02)	−0.04* (0.01)	0.01*** (0.00)	0.01 (0.00)	0.00 (0.00)
Wealth index (proxy of permanent income)	−0.08** (0.03)	−0.08 (0.05)	−0.07 (0.06)	−0.02 (0.01)	−0.04** (0.02)	−0.03* (0.01)
Observations	11 471	11 471	11 471	1560	1560	1560
Link test: coefficient of square of the predicted values				0.88	−0.14	−0.07
P value				0.42	0.52	0.43
Correlation among residuals of part 1 and part 2				−0.01	0.22	0.15

*, ** and *** indicate significance at 10%, 5% and 1% levels, respectively. SEs are in parentheses. The estimation controlled for individual sociodemographic and economic characteristics, different tobacco-related warnings, advertising, promotional initiatives, and perceptions related to smoking and tobacco taxes. The corresponding marginal effects are provided in online supplemental table 1. For the link test, the insignificance of coefficients of the square of the predicted values indicates the parsimonious nature of the specifications.

BDT, Bangladeshi taka; GATS, Global Adult Tobacco Survey.

The sign of prevalence elasticity estimates for biri price was negative, establishing the complementarity of biri for cigarette smoking decisions. Although the magnitude is negligible, its significance confirmed that biri price is an important determinant of cigarette smoking decisions. Conversely, intensity elasticity estimates of biri price were positive, indicating the substitutability of cigarettes and biris for smoking intensity. However, since this effect is statistically insignificant, biri price might not be a key determinant of the intensity of cigarette smoking. Therefore, biris can be considered as weak substitute for cigarettes, and most smokers do not tend to switch to biris in response to an increase in cigarette price. The magnitude of proxy income elasticity (wealth index proxied permanent income) is relatively small, and it is statistically significant only in model 1 (for prevalence) and in models 2 and 3 (for intensity).

### Restricting data by wealth group

The sample was divided into two wealth groups, designating the top two wealth quintiles as the high-wealth group and bottom three as the low-wealth. Table 2 contains the elasticity estimates, and the marginal effects are given in online supplemental tables 2 and 3. Across wealth groups, cigarette price negatively affected smoking prevalence and intensity. The impact was higher in the low-wealth group than in the high-wealth group, irrespective of model choice. The prevalence elasticity ranged from −0.58 to −0.88 in the low-wealth group, while in the high-wealth group, it spanned between −0.30 and −0.37. The difference in the elasticity coefficients was statistically significant (test statistic values were 127.91, 139.81 and 136.34, respectively, for models 1, 2 and 3 with p<0.05 for all). The reason for the lower responsiveness in the high-wealth group may reflect their higher ability to pay. As before, the magnitudes of the intensity elasticity estimates were small and were found to be significant for the low-wealth group only in model 1 and for the high-wealth group in models 1 and 2.

**Table 2:** Prevalence and intensity elasticity of cigarette smoking across wealth groups

Variables	Prevalence elasticities			Intensity elasticities		
	Model 1	Model 2	Model 3	Model 1	Model 2	Model 3
**Low-wealth group (60%)**						
Average consumption-weighted cigarette price (BDT per stick)	−0.58*** (0.11)	−0.84*** (0.20)	−0.88*** (0.20)	−0.08* (0.05)	−0.05 (0.05)	−0.04 (0.05)
Average consumption-weighted biri price (BDT per stick)	0.01 (0.01)	0.01 (0.02)	0.00 (0.02)	0.00 (0.00)	0.00 (0.00)	0.00 (0.00)
Wealth index (proxy of permanent income)	−0.17** (0.08)	−0.03 (0.15)	0.00 (0.16)	0.02 (0.06)	0.01 (0.06)	0.01 (0.06)
Observations	7680	7680	7680	961	961	961
Link test: coefficient of square of the predicted values				5.67	−0.06	−0.13
P value				0.02	0.69	0.23
Correlation among residuals of part 1 and part 2				−0.03	0.07	0.07
**High-wealth group (40%)**						
Average consumption-weighted cigarette price (BDT per stick)	−0.30*** (0.11)	−0.35*** (0.13)	−0.37*** (0.14)	−0.05* (0.03)	−0.06** (0.03)	−0.04 (0.03)
Average consumption-weighted biri price (BDT per stick)	−0.08*** (0.03)	−0.10*** (0.04)	−0.12*** (0.04)	0.01** (0.01)	0.01 (0.01)	0.01 (0.01)
Wealth index (proxy of permanent income)	0.01 (0.12)	0.08 (0.11)	0.01 (0.11)	0.03 (0.04)	0.10* (0.05)	0.07 (0.05)
Observations	3791	2228	2228	599	599	599
Link test: coefficient of square of the predicted values				−0.07	−0.32	−0.08
P value				0.94	0.14	0.25
Correlation among residuals of part 1 and part 2				0.01	0.01	0.06

*, ** and *** indicate significance at 10%, 5% and 1% levels, respectively. SEs are in parentheses. The estimation attempts also controlled for different tobacco-related warnings, advertising, promotional initiatives, and perceptions related to smoking and tobacco taxes. The corresponding marginal effects are given in online supplemental tables 2 and 3. For the link test, the insignificance of coefficients of the square of the predicted values indicates the parsimonious nature of the specifications.

BDT, Bangladeshi taka.

Negative prevalence elasticity estimates for biri price suggested its complementarity for smokers in the high-wealth group when deciding to smoke cigarettes. However, biri price is mostly insignificant and had a negligible impact on the smoking intensity regardless of the wealth group. Similarly, within each wealth group, wealth index also had a statistically insignificant impact on cigarette smoking prevalence and intensity.

### Restricting data by residential group

Cigarette smoking behaviour can differ according to urban–rural residential status. The regional estimates of price elasticity are presented in table 3 (corresponding marginal effects are given in online supplemental tables 4 and 5).

**Table 3:** Prevalence and intensity elasticity of cigarette smoking across residential areas

Variables	Prevalence elasticities			Intensity elasticities		
	Model 1	Model 2	Model 3	Model 1	Model 2	Model 3
**Rural**						
Average consumption-weighted cigarette price (BDT per stick)	−0.44*** (0.10)	−0.68*** (0.16)	−0.70*** (0.17)	−0.04 (0.03)	−0.03 (0.03)	−0.01 (0.03)
Average consumption-weighted biri price (BDT per stick)	0.00 (0.01)	−0.02 (0.02)	−0.03* (0.03)	0.01** (0.01)	0.02** (0.01)	0.01 (0.01)
Wealth index (proxy of permanent income)	−0.08*** (0.03)	−0.10 (0.07)	−0.08 (0.07)	−0.03 (0.03)	−0.09*** (0.03)	−0.06** (0.03)
Observations	7139	7139	7139	868	868	868
Link test: coefficient of square of the predicted values				0.69	−0.05	−0.03
P value				0.77	0.82	0.67
Correlation among residuals of part 1 and part 2				−0.03	0.04	0.05
**Urban**						
Average consumption-weighted cigarette price (BDT per stick)	−0.36*** (0.09)	−0.63*** (0.15)	−0.52*** (0.17)	−0.09* (0.05)	−0.11** (0.05)	−0.10** (0.04)
Average consumption-weighted biri price (BDT per stick)	−0.04* (0.02)	−0.07* (0.04)	−0.04 (0.04)	0.00 (0.00)	0.00 (0.00)	0.00 (0.00)
Wealth index (proxy of permanent income)	−0.01 (0.03)	−0.03 (0.04)	−0.05 (0.04)	0.01 (0.01)	0.00 (0.01)	0.00 (0.01)
Observations	4332	4332	4332	692	692	692
Link test: coefficient of square of the predicted values				3.08	−0.25	−0.18
P value				0.15	0.39	0.23
Correlation among residuals of part 1 and part 2				−0.01	0.05	0.10

*, ** and *** indicate significance at 10%, 5% and 1% levels, respectively. SEs are in parentheses. The estimation attempts also controlled for different tobacco-related warnings, advertising, promotional initiatives, and perceptions related to smoking and tobacco taxes. The corresponding marginal effects are given in online supplemental tables 4 and 5. For the link test, the insignificance of coefficients of the square of the predicted values indicates the parsimonious nature of the specifications.

BDT, Bangladeshi taka.

The price elasticity of cigarette smoking prevalence was higher in rural areas (−0.44 to −0.70) than in urban areas (−0.36 to −0.52). Similar to the prevalence of cigarette smoking, smoking intensity was also negatively affected regardless of area of residence. Nevertheless, this negative impact on cigarette smoking intensity was statistically significant and more prominent among smokers from urban areas than those from rural areas. It might be driven by greater variation in cigarette prices in urban areas. Although small in magnitude, biri price had a significant and complementary effect on cigarette smoking prevalence in both rural and urban areas. Conversely, it showed a negligible effect on cigarette smoking intensity in urban areas. Wealth was an insignificant factor in urban areas for predicting the prevalence and intensity of cigarette smoking. In rural areas, it was occasionally significant, with a small magnitude of effect on the outcome of prevalence and intensity.

### Total cigarette price elasticity

Total elasticity estimates were given by the sum of prevalence and intensity elasticity estimates. Table 4 contains the results.

**Table 4:** Total cigarette price elasticity using GATS data

Total cigarette price elasticity	Model 1	Model 2	Model 3
Overall	−0.51	−0.71	−0.73
Low-wealth group (lowest 60%)	−0.66	−0.89	−0.92
High-wealth group (highest 40%)	−0.35	−0.41	−0.41
Rural area of residence	−0.48	−0.71	−0.71
Urban area of residence	−0.45	−0.74	−0.62

GATS, Global Adult Tobacco Survey.

The price elasticity of cigarette demand varied between −0.51 and −0.73 in different specifications. Estimation in model 3 was the most accurate, as it additionally controlled for tobacco-related covariates. Therefore, it can be asserted that a 10% increase in cigarette price would lead to, on average, a 7.3% fall in cigarette demand in Bangladesh. The cigarette demand, therefore, responds less than proportionately to the cigarette price and can be considered price inelastic. Additionally, since the magnitude of elasticity remained less than one (in absolute terms) for all models and in both wealth groups, it is inelastic regardless of people’s wealth status. Considering model 3 for the high-wealth group, a 10% increase in cigarette price would lead to a decrease in cigarette demand of 4.1%, while the same percentage of price change would bring a reduction in cigarette demand as high as 9.2% in the low-wealth group.

This finding is of crucial importance in the context of Bangladesh, particularly because of the high prevalence of tobacco smoking in the lower wealth quintiles (48.8% and 26.3% in the lowest and highest wealth quintile, respectively).^1^ Thus, an increase in cigarette price would be more effective in reducing smoking prevalence in low-wealth groups. An estimation, taking into account the areas of residence, showed that the magnitude of total cigarette price elasticity is marginally higher in rural areas than in urban ones. However, cigarette demand was price inelastic regardless of the area of residence. In rural areas, the elasticity varied between −0.48 and −0.71, while in urban, it ranged between −0.45 and −0.62. Relative income differences could be the underlying reason for this differential impact. People living in rural areas generally have lower incomes and are therefore more responsive to price changes than those in urban areas.

### Research limitations

Smokeless tobacco, which is a substitute for cigarettes, has a prevalence of 20.6% in Bangladesh.^1^ The estimated models ignored the smokeless tobacco price as a covariate due to the unavailability of appropriate data in both rounds of GATS. Since a nationally representative survey with regional prices for cigarettes was unavailable, the scope of the robustness check for alternative price data was limited. In the absence of a valid instrument for self-reported prices, the consumption-weighted cluster average prices were used, as the cluster average price reflects the average price level that individuals face in the market. There were 496 clusters in the data that were observed in both survey years, which gave enough data points on cigarette price to offer sufficient cross-sectional and time variation in prices to estimate the effect of a price increase on smoking behaviour. It is an accepted practice to merge individual-level data with community/subnational/national level variables, as individuals living in adjacent geographical areas share similar characteristics with the neighbouring communities.^33 34^ Even if there are concerns that this approach may introduce measurement errors by using cluster-level prices as opposed to individual-level self-reported prices, it has the potential to minimise the endogeneity of the self-reported price variable and thus trade off measurement error bias for endogeneity bias.

Given the absence of individual income in GATS, a wealth index (proxied for income) was controlled for in the cigarette demand models. Owing to the limited number of observations in the datasets, age-specific and gender-specific elasticity estimations could not be performed. The launch of novel tobacco products and the repositioning of a few existing brands, along with the outbreak of the COVID-19 pandemic, might have impacted smokers’ behaviour in recent years.^56 57^ Furthermore, a systematic rather than a sequential estimation of the two-part model can be performed to examine the coefficients’ variation. Further research is required with more recent data and methods.

## Discussion and policy recommendations

The results revealed cigarette demand to be price inelastic and to range between −0.51 and −0.73, consistent with previous estimates by Nargis *et al*.^34^ Though it was also price inelastic across all socioeconomic and demographic groups in Bangladesh, it did not differ considerably across geographical locations. Individuals in the low-wealth group were more than twice as responsive to price change than their high-wealth counterparts. Thus, the relationship between cigarette price and its demand is inverse across socioeconomic and demographic groups. Most importantly, the greater magnitude of the elasticity parameter for the low-wealth group implies that, in response to a price increase, members of poorer households will quit cigarette smoking more readily than the rich. The price elasticity of cigarettes increased when all possible tobacco-control covariates were controlled for, as they can potentially shift the cigarette demand function and alter the estimates. It indicates that people are more likely to respond to a change in cigarette prices by quitting smoking in a comprehensive tobacco-control environment. The finding is plausible, as it indicates that in addition to price, the non-tax covariates are also important for smoking decisions and make people more responsive when those are controlled for along with cigarette price.

Although the general conclusions are similar to those in the existing studies from Bangladesh, the magnitude of the estimates differs.^33–36^ The study estimated total price elasticity, which is larger in absolute values than previous estimates with a greater contribution from prevalence elasticity. This is in contrast to the findings from high-income countries where the average share of prevalence elasticity is half, but corresponds to findings from LMICs.^5 16 46 58^ In addition to differences in method and the more recent data from 2017 used for the analysis, knowledge and perception about the adverse health impact of cigarette smoking have improved over time, which has made people more responsive to cigarette price increases. The relatively greater contribution of prevalence elasticity over intensity elasticity may reflect the fact that reduction in smoking initiation is playing a more important role in the total reduction in cigarette consumption than smoking cessation. While smoking cessation is a slower process, reduction in smoking initiation directly implies reduction in smoking participation, accounting for a major proportion of the sensitivity in total cigarette consumption.

The study estimated the negative impact of wealth index (proxy for income) on smoking, although this was mostly insignificant. While it is expected that income will have a positive impact on smoking, it is not unusual to find a negative impact.^16 59 60^ In LMICs experiencing rapid income growth, knowledge and perception about the health risks of smoking are likely to improve significantly with better healthcare systems. While income growth is commonly expected to spur demand, the mediation of the advancement in health-related indicators in a growing income setting would be reflected in a negative relation between income growth and cigarette smoking. We believe that this is the underlying reason driving the negative sign of the coefficient for the wealth index. For instance, a negative income elasticity for smoking in Japan, in rural areas in Myanmar and more recently for low-price cigarettes in Bangladesh was estimated.^16 59 60^ Thus, the negative effect of an increase in income on smoking intensity can actually be larger in some instances than the positive effect of income on smoking prevalence.

The negative prevalence elasticity of cigarettes demonstrates that increasing cigarette taxes reduces smoking prevalence. Provided there is sufficient pass-through of the tax to cigarette prices, inelasticity ensures that the tax burden falls more on smokers. In other words, the lower the cigarette demand responsiveness is, the greater the industry’s incentive to pass on tax increases, and hence, the larger is the tax burden on smokers. However, since the low-wealth group is more responsive than the high-wealth group, the relative tax burden would fall more on smokers from the high-wealth group. The number of smokers would decrease as a result, but there would be an increase in government revenue.^32 45 61^ A considerable number of studies established the health benefits (measured by the number of lives saved) achieved through taxation on cigarettes, assuming cigarettes to be price inelastic.^62–65^ Given the price inelasticity of cigarettes, there has also been sufficient evidence that a cigarette tax increases government revenue.^43 66–68^ Thus, the findings highlight and reinforce the importance of cigarette taxation for achieving the twin policy goals of improving public health and increasing government tax revenue.

The cigarette market in Bangladesh changed dramatically over the last decade, with massive growth in low-price cigarette sales and a consequent increase in their market share.^69^ There had been brand substitution from higher-price to lower-price cigarettes due to widening price differential between brands, which was attributable to a differential four-tiered cigarette tax structure with a significantly lower tax rate for low-price brands. Despite tax and price increases, this downward substitution resulted in a constant rate of cigarette smoking among adults, rather than quitting smoking. This large market share for lower-price cigarettes, combined with the growth of per capita income and a change in preference of the former biri smokers, also led to product substitution from biri to low-price cigarettes.^36^ The results of our study suggest that a substantial increase in cigarette prices at the low tier would effectively reduce overall cigarette consumption.

For the biri price, the study found negative prevalence elasticity and positive intensity elasticity, but the effect was statistically insignificant. A similar complementary effect of biri price was also found in India.^3^ Given the biri features in Bangladesh (hand rolled in white paper and look like cigarettes), smokers can switch between biris and cigarettes and use both depending on the changes in prices and affordability, along with other determinants of demand in general. The insignificance of the biri price could be due to the low base price for biris as well as the low prevalence of biri smoking. The prevalence of overall biri smoking in the country is around 5%, and a pack of biri with 25 sticks is sold at a price of around 12.50 Bangladeshi taka.^1^ This implies that a percentage change in the base price for biris results in an insignificant incremental change in absolute actual price, which, in turn, fails to affect the intensity of cigarette smoking markedly. In addition, although the price gap between cigarettes and biris is wide, the high growth of per capita income made biris a less attractive choice for increasing smoking intensity. It can be argued here that biri is the lowest-cost substitute for cigarettes, although individuals appear to be less interested in them, perhaps because of an increase in income over the years or the social status (as biris are typically consumed by comparatively low-income people) associated with biri smoking.

The effectiveness of cigarette taxation is subject to the efficiency of the tax structure design and to proper implementation. In Bangladesh, the tobacco tax structure is complex and tiered, and prices of cigarettes are comparatively low. In order for taxation to effectively reduce prevalence, the tax structure can either be a mixed system, with a blend of specific and ad valorem tax such that the specific component constitutes the majority of the total tax incidence, or it can be a purely specific tax system. Moreover, the tax must be annually adjusted for inflation and income growth to maintain its effectiveness with regard to the real value of the cigarette price.

## Data Availability

Data are available in a public, open access repository. GATS 2009 and GATS 2017 datasets for Bangladesh are available in the Centers for Disease Control and Prevention (CDC), National Center for Chronic Disease Prevention and Health Promotion, Office of Smoking and Health, and Global Tobacco Surveillance System Data (GTSSData) URL: https://www.cdc.gov/tobacco/global/gtss/gtssdata/index.html.
