# Supplementary material for: Price elasticity of cigarette smoking in Bangladesh: evidence from the Global Adult Tobacco Surveys (GATS)
Source: Tob Control. 2023 Aug 8;33(Suppl 2):s51–8. doi: 10.1136/tc-2022-057668 (PMC11187381; doi:10.1136/tc-2022-057668)
Supplement: Supplementary data [file tc-2022-057668supp001.pdf]

Wealth Index Calculation by Principal Component Analysis (PCA)

Step 1: First we choose the following variables i.e. wealth items (considering their availability in GATS 2009 and GATS 2017 Data) to calculate the wealth index:

Electricity, Toilet (Flush Toilet), Phone (Cell/Fixed), Television, Radio, Refrigerator, Car, Motorcycle (Moped/Scooter) Washing Machine, Bicycle, Sewing Machine, Almirah (Wardrobe) Table, Bed (Cot), Clock (Watch), Chair (Bench)

Step 2: Convert all the variables into binary where 1=presence of the wealth and 0=absence of the wealth.

Step 3: We performed PCA separately for rural and urban group. Since weight of each wealth items (variables) will be different for rural and urban group. The table below contains the scoring coefficients:

Scoring Coefficients for the Wealth Index																
**Wealth**	**Components**															
	**1**	**2**	**3**	**4**	**5**	**6**	**7**	**8**	**9**	**10**	**11**	**12**	**13**	**14**	**15**	**16**
**Electricity**	0.23	-0.08	-0.54	-0.06	-0.10	0.30	0.17	0.25	-0.05	0.23	0.14	0.49	-0.20	0.26	-0.17	0.05
**Toilet**	0.22	0.46	0.02	-0.14	0.04	-0.08	-0.17	-0.07	-0.32	-0.50	0.36	0.36	0.13	0.02	0.25	0.02
**Phone**	0.28	-0.15	-0.37	0.26	-0.09	0.17	-0.15	-0.11	-0.04	-0.04	-0.02	-0.22	0.76	0.01	-0.03	-0.05
**Television**	0.35	-0.05	-0.26	-0.12	-0.04	0.11	0.10	0.15	0.02	0.01	0.03	-0.28	-0.26	-0.62	0.46	-0.02
**Radio**	0.07	0.12	0.37	-0.09	-0.05	0.83	-0.28	-0.12	0.17	0.07	0.11	-0.05	-0.05	-0.01	-0.05	0.01
**Refrigerator**	0.36	0.18	-0.11	-0.12	0.17	-0.12	-0.08	-0.04	0.01	-0.20	0.08	-0.32	-0.21	-0.12	-0.75	-0.02
**Car**	0.08	0.43	0.01	0.39	-0.38	-0.07	0.14	0.24	0.61	-0.20	-0.09	0.04	-0.01	0.04	0.02	-0.02
**Motorcycle**	0.18	0.17	0.02	0.06	0.59	-0.15	-0.48	0.34	0.24	0.34	-0.12	0.13	0.08	0.02	0.12	0.02
**Washing Machine**	0.11	0.53	0.11	0.25	-0.18	-0.01	0.15	-0.03	-0.51	0.53	-0.12	-0.15	0.00	-0.02	-0.02	0.01
**Bicycle**	0.09	-0.25	0.27	0.54	0.29	0.18	0.23	0.44	-0.28	-0.29	0.12	-0.08	-0.09	0.06	-0.02	0.00
**Sewing Machine**	0.18	0.08	0.01	0.17	0.48	0.06	0.51	-0.58	0.24	0.08	0.05	0.15	0.02	-0.02	0.08	0.01
**Almirah**	0.36	-0.03	0.01	-0.18	-0.01	-0.04	0.01	-0.06	0.01	-0.07	-0.14	-0.41	-0.19	0.71	0.31	0.00
**Table**	0.33	-0.26	0.21	0.19	-0.22	-0.18	-0.20	-0.19	0.00	0.12	0.09	0.23	-0.16	-0.04	-0.01	-0.70
**Bed**	0.22	-0.08	0.37	-0.39	-0.09	-0.15	0.35	0.30	0.15	0.25	0.43	-0.04	0.37	0.00	-0.06	0.03
**Chair**	0.32	-0.27	0.19	0.23	-0.23	-0.19	-0.22	-0.21	0.02	0.08	0.06	0.15	-0.12	-0.07	-0.01	0.71
**Clock**	0.30	-0.05	0.23	-0.25	-0.02	0.08	0.17	0.09	-0.10	-0.18	-0.75	0.30	0.16	-0.12	-0.09	0.00

Step 4: We predict (generate) the wealth index for rural and urban group separately using the scoring coefficients and combined them to have the wealth index. The summary statistics of the index is as follows:

Variable	Observations	Mean	Std. Deviation	Minimum	Maximum
**Wealth Index**	22,411	-0.64	2.02	-5.47	5.01

Step 6: Finally, we created the wealth quintiles. The distribution is given below:

Wealth Index	Frequency	Percent
**Very Low**	4,497	20.07
**Low**	4,471	19.95
**Medium**	4,620	20.61
**High**	5,380	24.01
**Very High**	3,443	15.36
**Total**	22,411	100.00

Step 7: Kaiser – Meyer – Olkin (KMO) measure of sampling adequacy was examined. The results are given below. Since the overall measure of KMO is greater than 0.5, the PCA approach is justified.

Variable	Kaiser - Meyer - Olkin (KMO) Measure
**Electricity**	0.83
**Toilet**	0.82
**Phone**	0.87
**Television**	0.88
**Radio**	0.78
**Refrigerator**	0.86
**Car**	0.71
**Motorcycle**	0.88
**Washing Machine**	0.72
**Bicycle**	0.75
**Sewing Machine**	0.91
**Almirah**	0.90
**Table**	0.81
**Bed**	0.86
**Chair**	0.80
**Clock**	0.90
**Overall**	0.85

Table S1: Marginal Effects for Part 1 and Part II estimations for Cigarette Smoking

	Part 1			Part 2		
**Variables**	**Model 1**	**Model 2**	**Model 3**	**Model 1**	**Model 2**	**Model 3**
**Dependent Variable**	**Smoking Status, 1 = Smoker and 0 = Non-Smoker**			**Ln(Cigarette Consumption)**		
Consumption Weighted Cigarette Price (Per Sticks)	-0.05***	-0.04***	-0.03***	-0.08**	-0.06	-0.05
Consumption Weighted Bidi Price (Per Sticks)	-0.00*	-0.00***	-0.00***	0.01***	0.01	0.01
Asset (Proxy of Permanent Income)	0.01***	0.00*	0.00	0.02	0.04**	0.03*
Year (2017=1, 2009=0)	0.04***	0.03***	0.03***	0.04	0.04	0.10
Female		-0.31***	-0.27***		-0.58	-0.59
Age		0.00	0.00***		0.00**	0.01***
Rural		-0.01	-0.01		-0.17***	-0.16**
Family Size		-0.00	-0.00		0.01	0.01
Education (Base Category: No Formal Education)						
Primary		-0.02**	-0.02**		-0.07	-0.04
Less than Secondary		-0.03***	-0.04***		-0.11	-0.09
Secondary Completed		-0.04**	-0.05***		-0.45***	-0.41***
High School Completed		-0.03	-0.03*		-0.55***	-0.57***
Bachelor		-0.00	-0.01		-0.45**	-0.42**
Post-Graduation		-0.09**	-0.09***		-1.02***	-0.92**
Employment (Base Category: Retired and Unemployed)						
Service		0.03	0.02		0.19	0.18
Business		0.05***	0.04**		0.39***	0.39***
Farming and Agricultural Worker		0.01	0.01		0.08	0.08
Industrial Worker		0.05**	0.06**		0.07	0.12
Daily Laborer		0.04**	0.03*		0.21	0.22
Self Employed		0.08***	0.07***		0.36**	0.32**
Student		-0.13***	-0.12***		-0.30	-0.23
Homemaker		0.01	0.01		0.01	0.18
Others		0.04*	0.03		0.30	0.32*
Household Smoking Not Allowed			-0.05***			-0.34***
Workplace Smoking Not Allowed			-0.01			-0.02
Warning						
Have Seen Newspaper Warning			0.01			0.08
Have Seen Magazine Warning			-0.04			-0.12
Have Seen TV Warning			0.00			-0.01
Have Listened to Radio Warning			0.00			0.02
Have Seen Billboard Warning			0.01			0.01
Have Seen Poster Warning			-0.02**			0.03
Have Seen Pack Warning			0.11***			0.23
Advertising						
Have Seen Store Advertising			0.01			0.13**
Have Seen TV Advertising			-0.01			0.08
Have Listened to Radio Advertising			0.07*			-0.26
Have Seen Billboard Advertising			0.01			0.00
Have Seen Poster Advertising			0.01			-0.08
Have Seen Newspaper Advertising			0.00			-0.18
Have Seen Magazine Advertising			0.10			-1.59***
Have Seen Cinema Advertising			-0.06**			-0.04
Have Seen Internet Advertising			-0.03			0.48
Have Seen Public Transport Advertising			0.00			0.06
Have Seen Public Walls Advertising			-0.03**			-0.09
Promotional Activities						
Have Seen Promotion through Sports			-0.06			-0.45
Have Seen Promotion through Arts and Culture Activities			-0.04			0.17
Have Seen Promotion through Movies			0.02**			0.12**
Have seen Free Sample Distribution			0.02			0.05
Have Seen Sold at Sale			0.02			0.12
Have Seen Coupon Distribution			-0.03*			0.06
Have Seen Special Discount			0.01			0.12
Have Seen Branding through Cloth			0.01			0.21**
Have Seen Promotion through Internet			-0.09			0.
Perception						
Second Hand Smoking Cause Illness			-0.03*			0.11
Smoking Causes Serious Illness			0.00			-0.29**
Smoking is Addictive			-0.01			0.08
In Favor of Tobacco Tax Increase			-0.04***			-0.07
Observations	11,471	11,471	11,471	1,560	1,560	1,560

Note: *, **, and *** indicate significance at 10%, 5%, and 1% levels, respectively.

Table S2: Marginal Effects for Part 1 and Part II estimations for Cigarette Smoking in Low Wealth Group (Low 60%)

	Part 1			Part 2		
**Variables**	**Model 1**	**Model 2**	**Model 3**	**Model 1**	**Model 2**	**Model 3**
**Dependent Variable**	**Smoking Status, 1 = Smoker and 0 = Non-Smoker**			**Ln(Cigarette Consumption)**		
Consumption Weighted Cigarette Price (Per Sticks)	-0.06***	-0.04***	-0.04***	-0.12*	-0.07	-0.06
Consumption Weighted Bidi Price (Per Sticks)	0.00**	0.00	-0.00	0.01	0.01	0.01
Asset (Proxy of Permanent Income)	0.01**	0.00	0.00	-0.01	-0.00	-0.01
Year (2017=1, 2009=0)	0.04***	0.03***	0.03***	-0.01	0.02	0.09
Female		-0.29***	-0.26***		0.10	-0.09
Age		0.00	0.00**		0.00	0.00
Rural		-0.02*	-0.01		-0.26***	-0.20***
Family Size		-0.00	-0.00		0.02	0.01
Education (Base Category: No Formal Education)						
Primary		-0.02**	-0.02**		-0.11	-0.10
Less than Secondary		-0.02*	-0.03**		-0.13	-0.12
Secondary Completed		-0.07***	-0.07***		-0.54**	-0.50**
High School Completed		-0.03	-0.01		-1.26***	-1.22***
Bachelor		-0.01	-0.04		-0.18	-0.32
Post Graduation		-0.17***	-0.16***		-1.29***	-1.30***
Employment (Base Category: Retired and Unemployed)						
Service		0.06**	0.06**		0.29	0.27
Business		0.08***	0.07***		0.53**	0.56***
Farming and Agricultural Worker		0.03	0.04*		0.16	0.18
Industrial Worker		0.09***	0.10***		0.24	0.32
Daily Laborer		0.05**	0.05**		0.28	0.30
Self Employed		0.10***	0.10***		0.58***	0.61***
Student		-0.15***	-0.13***		-0.59	-0.80*
Homemaker		0.05	0.05		-0.42	-0.17
Others		0.04	0.05		0.45*	0.47**
Household Smoking Not Allowed			-0.05***			-0.25***
Workplace Smoking Not Allowed			-0.01			-0.05
Warning						
Have Seen Newspaper Warning			0.01			0.02
Have Seen Magazine Warning			-0.07**			0.01
Have Seen TV Warning			0.01			0.02
Have Listened to Radio Warning			-0.01			-0.06
Have Seen Billboard Warning			0.00			0.08
Have Seen Poster Warning			-0.02*			0.05
Have Seen Pack Warning			0.09***			0.19
Advertising						
Have Seen Store Advertising			-0.00			0.09
Have Seen TV Advertising			-0.00			0.37*
Have Listened to Radio Advertising			0.07			0.22
Have Seen Billboard Advertising			0.00			0.18
Have Seen Poster Advertising			0.03**			-0.02
Have Seen Newspaper Advertising			-0.04			-1.65***
Have Seen Magazine Advertising			0.11			1.18
Have Seen Cinema Advertising			-0.07***			0.10
Have Seen Internet Advertising			-0.02			-0.16
Have Seen Public Transport Advertising			-0.02			-0.09
Have Seen Public Walls Advertising			-0.02			-0.24
Promotional Activities						
Have Seen Promotion through Sports			-0.15***			-0.40
Have Seen Promotion through Arts and Culture Activities			-0.00			0.18
Have Seen Promotion through Movies			0.02*			0.16**
Have seen Free Sample Distribution			0.03**			0.03
Have Seen Sold at Sale			-0.02			0.13
Have Seen Coupon Distribution			-0.01			0.02
Have Seen Special Discount			0.03*			0.15
Have Seen Branding through Cloth			0.03*			0.42***
Have Seen Promotion through Internet			0.03			-0.06
Perception						
Second Hand Smoking Cause Illness			-0.02			0.03
Smoking Causes Serious Illness			0.00			-0.28*
Smoking is Addictive			0.00			0.26**
In Favor of Tobacco Tax Increase			-0.04***			-0.11
Observations	7,680	7,680	7,680	961	961	961

Note: *, **, and *** indicate significance at 10%, 5%, and 1% levels, respectively.

Table S3: Marginal Effects for Part 1 and Part II estimations for Cigarette Smoking in High Wealth Group (High 60%)

	Part 1			Part 2		
**Variables**	**Model 1**	**Model 2**	**Model 3**	**Model 1**	**Model 2**	**Model**
**Dependent Variable**	**Smoking Status, 1 = Smoker and 0 = Non-Smoker**			**Ln(Cigarette Consumption)**		
Consumption Weighted Cigarette Price (Per Sticks)	-0.04***	-0.05***	-0.04***	-0.07*	-0.08**	-0.05
Consumption Weighted Bidi Price (Per Sticks)	-0.02***	-0.02***	-0.02***	0.03**	0.03	0.02
Asset (Proxy of Permanent Income)	0.00	0.01	0.00	0.03	0.11*	0.08
Year (2017=1, 2009=0)	0.04**	0.04	0.04	0.13	0.11	0.18*
Female		-0.55***	-0.50***		-1.98***	-
Age		0.00	0.00		0.01**	0.01**
Rural		-0.01	-0.02		-0.01	-0.06
Family Size		-0.00	-0.00		0.00	-0.00
Education (Base Category: No Formal Education)						
Primary		-0.03	-0.04		0.09	0.05
Less than Secondary		-0.07**	-0.10***		-0.02	0.00
Secondary Completed		-0.03	-0.07*		-0.37**	-0.35*
High School Completed		-0.06	-0.08**		-0.32	-0.36*
Bachelor		-0.00	-0.04		-0.45*	-0.35*
Post Graduation		-0.15**	-0.18***		-0.89**	-0.70
Employment (Base Category: Retired and Unemployed)						
Service		-0.00	-0.05		0.22	0.17
Business		0.03	-0.03		0.39*	0.38*
Farming and Agricultural Worker		0.00	-0.04		0.27	0.28
Industrial Worker		-0.03	-0.07		0.00	-0.06
Daily Laborer		0.13**	0.04		0.33	0.31
Self Employed		0.09	0.01		0.02	-0.29
Student		-0.22***	-0.22***		-0.15	0.00
Homemaker		-	-		-	-
Others		0.07	0.01		0.13	0.14
Household Smoking Not Allowed			-0.09***			-
Workplace Smoking Not Allowed			-0.03			-0.07
Warning						
Have Seen Newspaper Warning			0.02			0.16
Have Seen Magazine Warning			-0.05			-0.30
Have Seen TV Warning			-0.01			-0.15
Have Listened to Radio Warning			0.04			0.11
Have Seen Billboard Warning			0.02			-0.03
Have Seen Poster Warning			-0.04*			0.02
Have Seen Pack Warning			0.30***			0.39*
Advertising						
Have Seen Store Advertising			0.05**			0.19*
Have Seen TV Advertising			-0.01			-0.25
Have Listened to Radio Advertising			0.08			-0.75
Have Seen Billboard Advertising			0.03			-0.12
Have Seen Poster Advertising			-0.02			-0.23*
Have Seen Newspaper Advertising			0.03			0.61**
Have Seen Magazine Advertising			0.05			-
Have Seen Cinema Advertising			-0.10			-0.26
Have Seen Internet Advertising			-0.10			0.87***
Have Seen Public Transport Advertising			0.07**			0.36**
Have Seen Public Walls Advertising			-0.07**			-0.04
Promotional Activities						
Have Seen Promotion through Sports			0.23*			-0.23
Have Seen Promotion through Arts and Culture Activities			-0.12			0.12
Have Seen Promotion through Movies			0.02			0.06
Have seen Free Sample Distribution			-0.01			0.06
Have Seen Sold at Sale			0.10			-0.04
Have Seen Coupon Distribution			-0.08*			0.15
Have Seen Special Discount			0.01			0.17
Have Seen Branding through Cloth			-0.03			-0.17
Have Seen Promotion through Internet			-0.28**			0.48
Perception						
Second Hand Smoking Cause Illness			-0.11*			0.04
Smoking Causes Serious Illness			0.06			-0.45*
Smoking is Addictive			-0.08*			-0.18
In Favor of Tobacco Tax Increase			-0.06***			-0.02
Observations	3,791	2,228	2,228	599	599	599

Note: *, **, and *** indicate significance at 10%, 5%, and 1% levels, respectively.

Table S4: Marginal Effects for Part 1 and Part II estimations for Cigarette Smoking in Rural Areas

	Part 1			Part 2		
**Variables**	**Model 1**	**Model 2**	**Model 3**	**Model 1**	**Model 2**	**Model 3**
**Dependent Variable**	**Smoking Status, 1 = Smoker and 0 = Non-Smoker**			**Ln(Cigarette Consumption)**		
Consumption Weighted Cigarette Price (Per Sticks)	-0.04***	-0.03***	-0.03***	-0.05	-0.03	-0.02
Consumption Weighted Bidi Price (Per Sticks)	-0.00	-0.00*	-0.00**	0.03**	0.04**	0.02
Asset (Proxy of Permanent Income)	0.01***	0.00*	0.00	0.03	0.08***	0.06**
Year (2017=1, 2009=0)	0.04***	0.03***	0.03***	0.02	-0.01	0.02
Female		-0.29***	-0.25***		-0.69	-0.67
Age		0.00	0.00**		0.00	0.00
Family Size		-0.00	-0.00		0.02	0.01
Education (Base Category: No Formal Education)						
Primary		-0.02	-0.02**		-0.11	-0.08
Less than Secondary		-0.04***	-0.04***		-0.24**	-0.24**
Secondary Completed		-0.04*	-0.04***		-0.57***	-
High School Completed		-0.05**	-0.04**		-0.53***	-
Bachelor		-0.01	-0.02		-0.74***	-
Post Graduation		-0.08*	-0.08**		-0.61	-0.24
Employment (Base Category: Retired and Unemployed)						
Service		0.03	0.02		0.12	0.12
Business		0.03*	0.02		0.46**	0.46**
Farming and Agricultural Worker		-0.00	0.00		0.09	0.09
Industrial Worker		0.03	0.03		0.03	0.12
Daily Laborer		0.02	0.01		0.26	0.27
Self Employed		0.05*	0.04**		0.55***	0.60***
Student		-0.14***	-0.13***		-0.62	-0.43
Homemaker		-0.02	-0.01		-0.32	-0.09
Others		0.02	0.01		0.37	0.45**
Household Smoking Not Allowed			-0.05***			-
Workplace Smoking Not Allowed			-0.02*			-0.01
Warning						
Have Seen Newspaper Warning			0.00			0.21*
Have Seen Magazine Warning			-0.04			0.42
Have Seen TV Warning			0.01			-0.03
Have Listened to Radio Warning			0.00			-0.12
Have Seen Billboard Warning			0.00			-0.09
Have Seen Poster Warning			-0.02**			0.12
Have Seen Pack Warning			0.11***			0.52**
Advertising						
Have Seen Store Advertising			0.02*			0.19**
Have Seen TV Advertising			-0.01			-0.14
Have Listened to Radio Advertising			0.08**			-0.09
Have Seen Billboard Advertising			0.00			0.07
Have Seen Poster Advertising			0.01			-0.05
Have Seen Newspaper Advertising			0.00			-1.50*
Have Seen Magazine Advertising			0.23***			0.38
Have Seen Cinema Advertising			-0.08**			0.32
Have Seen Internet Advertising			-0.07			0.17
Have Seen Public Transport Advertising			0.01			-0.04
Have Seen Public Walls Advertising			-0.04**			-0.29
Promotional Activities						
Have Seen Promotion through Sports			-0.12***			-
Have Seen Promotion through Arts and Culture Activities			-0.03			1.21**
Have Seen Promotion through Movies			0.02*			0.06
Have seen Free Sample Distribution			0.03**			0.04
Have Seen Sold at Sale			-0.04			0.09
Have Seen Coupon Distribution			-0.02			0.09
Have Seen Special Discount			0.01			0.03
Have Seen Branding through Cloth			0.03			0.51***
Have Seen Promotion through Internet			-0.02			0.01
Perception						
Second Hand Smoking Cause Illness			-0.04*			0.08
Smoking Causes Serious Illness			-0.00			-
Smoking is Addictive			-0.01			0.06
In Favor of Tobacco Tax Increase			-0.04***			-0.07
Observations	7,139	7,139	7,139	868	868	868

Note: *, **, and *** indicate significance at 10%, 5%, and 1% levels, respectively.

Table S5: Marginal Effects for Part 1 and Part II estimations for Cigarette Smoking in Urban Areas

	Part 1			Part 2		
**Variables**	**Model 1**	**Model 2**	**Model 3**	**Model 1**	**Model 2**	**Model 3**
**Dependent Variable**	**Smoking Status, 1 = Smoker and 0 = Non-Smoker**			**Ln(Cigarette Consumption)**		
Consumption Weighted Cigarette Price (Per Sticks)	-0.08***	-0.06***	-0.04***	-0.15*	-0.18**	-0.16**
Consumption Weighted Bidi Price (Per Sticks)	-0.01**	-0.01***	-0.00**	0.00	-0.00	-0.00
Asset (Proxy of Permanent Income)	0.01	0.00	0.01	-0.02	0.00	-0.01
Year (2017=1, 2009=0)	0.06	0.04	0.00	0.13	0.14	0.22**
Female		-0.45***	-0.40***		0.11	-0.07
Age		0.00	0.00*		0.01**	0.01**
Family Size		0.00	0.00		0.01	0.00
Education (Base Category: No Formal Education)						
Primary		-0.03	-0.03*		-0.02	0.04
Less than Secondary		-0.01	-0.02		0.05	0.09
Secondary Completed		-0.04	-0.05		-0.31*	-0.20
High School Completed		-0.01	-0.05		-0.58**	-0.56*
Bachelor		0.02	0.02		-0.16	-0.11
Post Graduation		-0.13***	-0.12***		-1.21**	-1.05*
Employment (Base Category: Retired and Unemployed)						
Service		0.08***	0.06**		0.23	0.34
Business		0.13***	0.10***		0.35	0.44*
Farming and Agricultural Worker		0.08**	0.06**		0.19	0.29
Industrial Worker		0.18***	0.17***		0.16	0.18
Daily Laborer		0.17***	0.14***		0.15	0.25
Self Employed		0.23***	0.16***		0.16	0.15
Student		-0.07*	-0.08**		0.13	0.09
Homemaker		0.16***	0.14***			
Others		0.14***	0.12**		0.25	0.32
Household Smoking Not Allowed			-0.07***			-
Workplace Smoking Not Allowed			0.01			-0.04
Warning						
Have Seen Newspaper Warning			-0.00			-0.00
Have Seen Magazine Warning			-0.01			-0.46*
Have Seen TV Warning			-0.02			0.05
Have Listened to Radio Warning			-0.03			0.19
Have Seen Billboard Warning			0.02			0.07
Have Seen Poster Warning			-0.00			-0.07
Have Seen Pack Warning			0.14***			-0.10
Advertising						
Have Seen Store Advertising			-0.02			0.05
Have Seen TV Advertising			0.01			0.22
Have Listened to Radio Advertising			0.05			0.49
Have Seen Billboard Advertising			0.07***			-0.05
Have Seen Poster Advertising			-0.01			-0.11
Have Seen Newspaper Advertising			-0.03			0.27
Have Seen Magazine Advertising			-0.16**			-
Have Seen Cinema Advertising			-0.07*			-0.10
Have Seen Internet Advertising			-0.01			0.87***
Have Seen Public Transport Advertising			-0.04			0.11
Have Seen Public Walls Advertising			0.01			0.07
Promotional Activities						
Have Seen Promotion through Sports			0.09			0.28
Have Seen Promotion through Arts and Culture Activities			-0.00			0.03
Have Seen Promotion through Movies			0.02			0.19**
Have seen Free Sample Distribution			0.02			0.11
Have Seen Sold at Sale			0.13***			0.09
Have Seen Coupon Distribution			-0.03			0.05
Have Seen Special Discount			0.03			0.24**
Have Seen Branding through Cloth			-0.04*			-0.02
Have Seen Promotion through Internet			-0.20**			0.64*
Perception						
Second Hand Smoking Cause Illness			-0.01			0.10
Smoking Causes Serious Illness			0.06			-0.09
Smoking is Addictive			-0.03			0.12
In Favor of Tobacco Tax Increase			-0.04***			-0.06
Observations	4,332	4,332	4,332	692	692	692

Note: *, **, and *** indicate significance at 10%, 5%, and 1% levels, respectively.

Separate Estimation of Prevalence and Intensity Elasticities with GATS 2009 and 2017 (For Overall Data)

Overall Data						
**Variables**	**GATS 2009**					
	**Prevalence Elasticities**			**Intensity Elasticities**		
	**Model 1**	**Model 2**	**Model 3**	**Model 1**	**Model 2**	**Model 3**
**Average Consumption Weighted Cigarette Price (BDT Per Stick)**	-0.43***<br>(0.09)	-0.65***<br>(0.19)	-0.72***<br>(0.20)	-0.13**<br>(0.06)	-0.08<br>(0.06)	-0.08<br>(0.06)
**Average Consumption Weighted Biri Price (BDT Per Stick)**	0.04<br>(0.03)	0.01<br>(0.05)	0.08<br>(0.05)	0.01<br>(0.04)	0.02<br>(0.04)	0.02<br>(0.05)
**Wealth Index (Proxy of Permanent Income)**	-0.04<br>(0.03)	0.02<br>(0.07)	0.00<br>(0.08)	0.00<br>(0.02)	-0.01<br>(0.02)	-0.02<br>(0.02)
**Observations**	5,282	5,282	5,278	687	687	687
**Link test: Coefficient of Square of the Predicted Values**				4.95	-0.02	-0.02
**P - Value**				0.00	0.91	0.92
**Variables**	**GATS 2017**					
	**Prevalence Elasticities**			**Intensity Elasticities**		
	**Model 1**	**Model 2**	**Model 3**	**Model 1**	**Model 2**	**Model 3**
**Average Consumption Weighted Cigarette Price (BDT Per Stick)**	-0.52***<br>(0.12)	-0.90***<br>(0.22)	-0.92***<br>(0.23)	-0.06*<br>(0.03)	-0.04<br>(0.03)	-0.03<br>(0.03)
**Average Consumption Weighted Biri Price (BDT Per Stick)**	-0.02<br>(0.02)	-0.06*<br>(0.03)	-0.08*<br>(0.04)	0.01***<br>(0.00)	0.01<br>(0.01)	0.01<br>(0.01)
**Wealth Index (Proxy of Permanent Income)**	-0.11**<br>(0.06)	-0.20**<br>(0.08)	-0.18**<br>(0.09)	-0.03*<br>(0.02)	-0.05**<br>(0.02)	-0.03<br>(0.02)
**Observations**	6,189	6,189	6,189	873	873	873
**Link test: Coefficient of Square of the Predicted Values**				0.60	-0.06	-0.04
**P - Value**				0.72	0.68	0.51

Note: *, **, and *** indicate significance at 10%, 5%, and 1% levels, respectively. Standard errors are in parentheses. The estimation controlled for individual socio-demographic and economic characteristics, different tobacco-related warnings, advertising, promotional initiatives, and perceptions related to smoking and tobacco taxes. BDT indicates Bangladeshi Taka. Under link test the insignificance of coefficients of square of the predicted values indicate the parsimonious nature of the specifications.

Separate Estimation of Prevalence and Intensity Elasticities with GATS 2009 and 2017 (For Low Wealth Group)

Low Wealth Group						
	GATS 2009					
Variables	Prevalence Elasticities			Intensity Elasticities		
	Model 1	Model 2	Model 3	Model 1	Model 2	Model 3
**Average Consumption Weighted Cigarette Price (BDT Per Stick)**	-0.53*** (0.13)	-0.73*** (0.25)	-0.68*** (0.26)	-0.20** (0.09)	-0.14 (0.09)	-0.10 (0.09)
**Average Consumption Weighted Biri Price (BDT Per Stick)**	0.06** (0.03)	0.08* (0.05)	0.13** (0.05)	-0.02 (0.05)	0.01 (0.05)	0.00 (0.06)
**Wealth Index (Proxy of Permanent Income)**	-0.15 (0.12)	0.16 (0.23)	0.17 (0.25)	0.03 (0.10)	-0.02 (0.11)	-0.03 (0.11)
**Observations**	3,404	3,307	3,305	399	399	399
**Link test: Coefficient of Square of the Predicted Values**				2.05	-0.02	-0.10
**P - Value**				0.07	0.89	0.43
	GATS 2017					
Variables	Prevalence Elasticities			Intensity Elasticities		
	Model 1	Model 2	Model 3	Model 1	Model 2	Model 3
**Average Consumption Weighted Cigarette Price (BDT Per Stick)**	-0.65*** (0.16)	-0.87*** (0.25)	-0.94*** (0.29)	-0.06 (0.06)	-0.01 (0.06)	-0.00 (0.07)
**Average Consumption Weighted Biri Price (BDT Per Stick)**	0.01 (0.01)	0.00 (0.03)	-0.02 (0.04)	0.01* (0.00)	0.01 (0.01)	0.01 (0.01)
**Wealth Index (Proxy of Permanent Income)**	-0.20** (0.10)	-0.25 (0.20)	-0.26 (0.24)	0.02 (0.07)	0.00 (0.07)	0.02 (0.08)
**Observations**	4,276	4,276	4,274	562	562	562
**Link test: Coefficient of Square of the Predicted Values**				4.25	-0.07	-0.10
**P - Value**				0.37	0.68	0.28

Note: *, **, and *** indicate significance at 10%, 5%, and 1% levels, respectively. Standard errors are in parentheses. The estimation controlled for individual socio-demographic and economic characteristics, different tobacco-related warnings, advertising, promotional initiatives, and perceptions related to smoking and tobacco taxes. BDT indicates Bangladeshi Taka. Under link test the insignificance of coefficients of square of the predicted values indicate the parsimonious nature of the specifications.

Separate Estimation of Prevalence and Intensity Elasticities with GATS 2009 and 2017 (For High Wealth Group)

High Wealth Group						
	**GATS 2009**					
**Variables**	**Prevalence Elasticities**			**Intensity Elasticities**		
	**Model 1**	**Model 2**	**Model 3**	**Model 1**	**Model 2**	**Model 3**
**Average Consumption Weighted Cigarette Price (BDT Per Stick)**	-0.30** (0.12)	-0.25** (0.12)	-0.43*** (0.16)	-0.06 (0.07)	-0.04 (0.07)	-0.03 (0.07)
**Average Consumption Weighted Biri Price (BDT Per Stick)**	-0.04 (0.06)	-0.08 (0.07)	-0.07 (0.11)	0.10 (0.07)	0.12* (0.07)	0.10 (0.07)
**Wealth Index (Proxy of Permanent Income)**	-0.17** (0.08)	-0.14 (0.10)	-0.15 (0.12)	0.00 (0.06)	0.02 (0.07)	0.02 (0.06)
**Observations**	1,878	870	868	288	288	288
**Link test: Coefficient of Square of the Predicted Values**				1.82	-0.21	-0.27
**P - Value**				0.52	0.39	0.04
	**GATS 2017**					
**Variables**	**Prevalence Elasticities**			**Intensity Elasticities**		
	**Model 1**	**Model 2**	**Model 3**	**Model 1**	**Model 2**	**Model 3**
**Average Consumption Weighted Cigarette Price (BDT Per Stick)**	-0.39** (0.16)	-0.49*** (0.19)	-0.51*** (0.20)	-0.07* (0.04)	-0.07 (0.04)	-0.05 (0.05)
**Average Consumption Weighted Biri Price (BDT Per Stick)**	-0.14*** (0.05)	-0.17*** (0.06)	-0.17*** (0.07)	0.02** (0.01)	0.02 (0.01)	0.00 (0.01)
**Wealth Index (Proxy of Permanent Income)**	0.18 (0.17)	0.34*** (0.12)	0.28*** (0.14)	0.06 (0.06)	0.11* (0.07)	0.06 (0.07)
**Observations**	1,913	1,154	1,148	311	311	311
**Link test: Coefficient of Square of the Predicted Values**				-0.24	-0.17	-0.05
**P - Value**				0.83	0.34	0.36

Note: *, **, and *** indicate significance at 10%, 5%, and 1% levels, respectively. Standard errors are in parentheses. The estimation controlled for individual socio-demographic and economic characteristics, different tobacco-related warnings, advertising, promotional initiatives, and perceptions related to smoking and tobacco taxes. BDT indicates Bangladeshi Taka. Under link test the insignificance of coefficients of square of the predicted values indicate the parsimonious nature of the specifications.

Separate Estimation of Prevalence and Intensity Elasticities with GATS 2009 and 2017 (For Rural Area)

Rural						
	GATS 2009					
Variables	Prevalence Elasticities			Intensity Elasticities		
	Model 1	Model 2	Model 3	Model 1	Model 2	Model 3
**Average Consumption Weighted Cigarette Price (BDT Per Stick)**	-0.40*** (0.10)	-0.62*** (0.20)	-0.69*** (0.23)	-0.08 (0.07)	-0.05 (0.07)	-0.08 (0.07)
**Average Consumption Weighted Biri Price (BDT Per Stick)**	0.03 (0.03)	0.01 (0.05)	0.07 (0.05)	0.03 (0.05)	0.03 (0.05)	0.05 (0.05)
**Wealth Index (Proxy of Permanent Income)**	-0.08 (0.04)	-0.01 (0.09)	-0.01 (0.11)	-0.04 (0.04)	-0.09** (0.04)	-0.09* (0.04)
**Observations**	3,145	3,145	3,141	346	346	346
**Link test: Coefficient of Square of the Predicted Values**				1.84	-0.18	-0.20
**P - Value**				0.62	0.45	0.14
**Rural**	GATS 2017					
Variables	Prevalence Elasticities			Intensity Elasticities		
	Model 1	Model 2	Model 3	Model 1	Model 2	Model 3
**Average Consumption Weighted Cigarette Price (BDT Per Stick)**	-0.52*** (0.14)	-0.89*** (0.24)	-1.04*** (0.27)	-0.03 (0.04)	-0.01 (0.04)	0.01 (0.04)
**Average Consumption Weighted Biri Price (BDT Per Stick)**	-0.01 (0.02)	-0.04 (0.04)	-0.08 (0.05)	0.02* (0.01)	0.02** (0.01)	0.01 (0.02)
**Wealth Index (Proxy of Permanent Income)**	-0.08** (0.04)	-0.19** (0.09)	-0.14 (0.11)	-0.03 (0.04)	-0.08* (0.04)	-0.03 (0.04)
**Observations**	3,994	3,994	3,994	522	522	522
**Link test: Coefficient of Square of the Predicted Values**				0.30	-0.05	-0.04
**P - Value**				0.92	0.77	0.47

Note: *, **, and *** indicate significance at 10%, 5%, and 1% levels, respectively. Standard errors are in parentheses. The estimation controlled for individual socio-demographic and economic characteristics, different tobacco-related warnings, advertising, promotional initiatives, and perceptions related to smoking and tobacco taxes. BDT indicates Bangladeshi Taka. Under link test the insignificance of coefficients of square of the predicted values indicate the parsimonious nature of the specifications.

Separate Estimation of Prevalence and Intensity Elasticities with GATS 2009 and 2017 (For Urban Area)

<table>
<tr><th colspan="7">Urban</th></tr>
<tr><th rowspan="3">Variables</th><th colspan="6">GATS 2009</th></tr>
<tr><th colspan="3">Prevalence Elasticities</th><th colspan="3">Intensity Elasticities</th></tr>
<tr><th>Model 1</th><th>Model 2</th><th>Model 3</th><th>Model 1</th><th>Model 2</th><th>Model 3</th></tr>
<tr><td>Average Consumption Weighted Cigarette Price (BDT Per Stick)</td><td>-0.49***
(0.16)</td><td>-0.84***
(0.29)</td><td>-0.98***
(0.32)</td><td>-0.16
(0.11)</td><td>-0.20*
(0.11)</td><td>-0.13
(0.10)</td></tr>
<tr><td>Average Consumption Weighted Biri Price (BDT Per Stick)</td><td>0.21**
(0.10)</td><td>0.36**
(0.19)</td><td>0.40*
(0.22)</td><td>-0.01
(0.12)</td><td>-0.01
(0.12)</td><td>-0.01
(0.12)</td></tr>
<tr><td>Wealth Index (Proxy of Permanent Income)</td><td>0.02
(0.01)</td><td>0.01
(0.04)</td><td>-0.04
(0.05)</td><td>0.03
(0.02)</td><td>0.02
(0.02)</td><td>0.03
(0.03)</td></tr>
<tr><td>Observations</td><td>2,137</td><td>2,137</td><td>2,137</td><td>341</td><td>341</td><td>341</td></tr>
<tr><td colspan="4">Link test: Coefficient of Square of the Predicted Values</td><td>2.71</td><td>-0.51</td><td>-0.31</td></tr>
<tr><td colspan="4">P - Value</td><td>0.01</td><td>0.01</td><td>0.01</td></tr>
<tr><th rowspan="3">Variables</th><th colspan="6">GATS 2017</th></tr>
<tr><th colspan="3">Prevalence Elasticities</th><th colspan="3">Intensity Elasticities</th></tr>
<tr><th>Model 1</th><th>Model 2</th><th>Model 3</th><th>Model 1</th><th>Model 2</th><th>Model 3</th></tr>
<tr><td>Average Consumption Weighted Cigarette Price (BDT Per Stick)</td><td>-0.43***
(0.13)</td><td>-0.41***
(0.13)</td><td>-0.20
(0.13)</td><td>-0.10
(0.07)</td><td>-0.12*
(0.06)</td><td>-0.07
(0.06)</td></tr>
<tr><td>Average Consumption Weighted Biri Price (BDT Per Stick)</td><td>-0.10**
(0.06)</td><td>-0.12*
(0.07)</td><td>-0.05
(0.08)</td><td>0.00
(0.00)</td><td>0.00
(0.01)</td><td>0.01
(0.01)</td></tr>
<tr><td>Wealth Index (Proxy of Permanent Income)</td><td>-0.07
(0.08)</td><td>-0.03
(0.03)</td><td>-0.03
(0.02)</td><td>-0.01
(0.01)</td><td>-0.02
(0.01)</td><td>-0.02
(0.01)</td></tr>
<tr><td>Observations</td><td>2,195</td><td>972</td><td>968</td><td>351</td><td>351</td><td>351</td></tr>
<tr><td colspan="4">Link test: Coefficient of Square of the Predicted Values</td><td>2.49</td><td>-0.28</td><td>-0.14</td></tr>
<tr><td colspan="4">P - Value</td><td>0.53</td><td>0.28</td><td>0.25</td></tr>
</table>

Note: *, **, and *** indicate significance at 10%, 5%, and 1% levels, respectively. Standard errors are in parentheses. The estimation controlled for individual socio-demographic and economic characteristics, different tobacco-related warnings, advertising, promotional initiatives, and perceptions related to smoking and tobacco taxes. BDT indicates Bangladeshi Taka. Under link test the insignificance of coefficients of square of the predicted values indicate the parsimonious nature of the specifications.
